# Improvement in Recurrent Laryngeal Nerve Paralysis and Tracheal Deviation after Surgical Resection of a Mediastinal Parathyroid Cyst: A Case Report

**DOI:** 10.70352/scrj.cr.24-0083

**Published:** 2025-01-31

**Authors:** Minoru Sugihara, Hideyuki Kaida, Mai Sugiura, Chihiro Hara, Yuriko Okazaki, Hisashi Yokoi, Sawako Okamoto, Hirofumi Takenaka, Tetsuo Taniguchi

**Affiliations:** 1 Department of Thoracic Surgery, Komaki City Hospital, Komaki, Aichi, Japan; 2 Department of Head and Neck Surgery, Komaki City Hospital, Komaki, Aichi, Japan

**Keywords:** mediastinal parathyroid cyst, recurrent laryngeal nerve paralysis, tracheal deviation

## Abstract

**INTRODUCTION:**

Mediastinal parathyroid cyst is a rare cystic disease that involves the parathyroid tissue within its walls. This case report is the first to document a mediastinal parathyroid cyst with recurrent laryngeal nerve paralysis and tracheal deviation that improved after surgical resection.

**CASE PRESENTATION:**

A 47-year-old man experienced hoarseness and dyspnea upon exertion for 1 month. Computed tomography revealed a mediastinal cystic lesion with a maximum diameter of 78 mm, compressing the trachea. Laryngofiberscopy suggested long-term left recurrent laryngeal nerve paralysis. Tumor resection was performed while preserving the left recurrent laryngeal nerve. The pathological examination led to the diagnosis of a mediastinal parathyroid cyst. Postoperatively, both tracheal deviation and recurrent laryngeal nerve paralysis improved.

**CONCLUSIONS:**

Surgical resection improved the tracheal deviation and recurrent laryngeal nerve paralysis caused by a mediastinal parathyroid cyst. Long-standing recurrent laryngeal nerve paralysis can improve, emphasizing the need for proactive surgical intervention and the importance of careful preservation of the recurrent laryngeal nerve.

## INTRODUCTION

Mediastinal parathyroid cyst is an uncommon cystic disease that involves the parathyroid tissue within its walls. It is usually asymptomatic and can rarely cause hoarseness due to recurrent laryngeal nerve paralysis, dysphagia, or tracheal compression-induced dyspnea.^1)^

This report presents the first case in which surgical resection of a mediastinal parathyroid cyst resulted in the improvement in both recurrent laryngeal nerve paralysis and tracheal deviation.

## CASE PRESENTATION

**Fig. 1:**
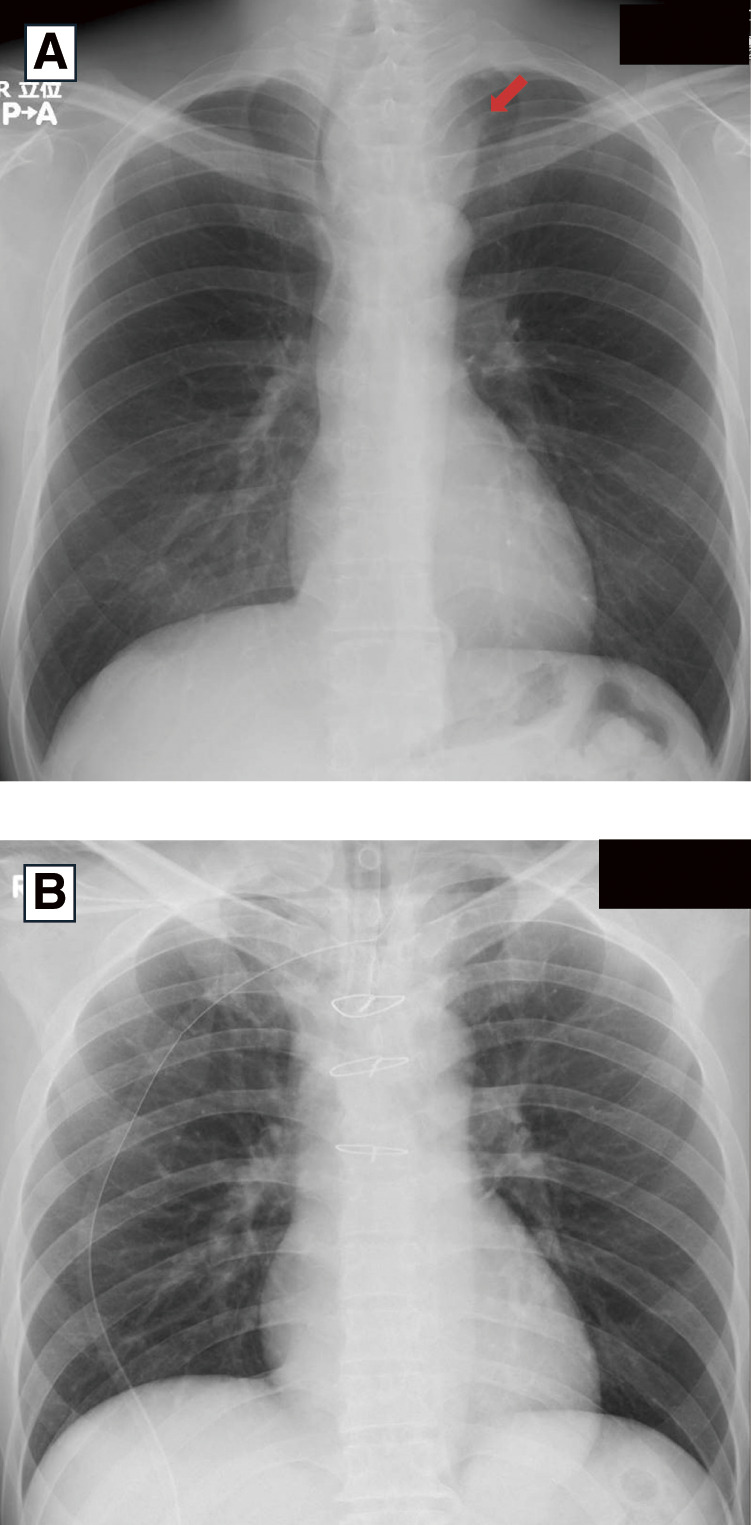
Chest radiography findings. (**A**) Chest radiography reveals a mass (arrow) on the left side of the trachea, which causes tracheal deviation to the right side of the trachea. (**B**) Postoperative chest radiograph showing improvement in tracheal deviation.

**Fig. 2:**
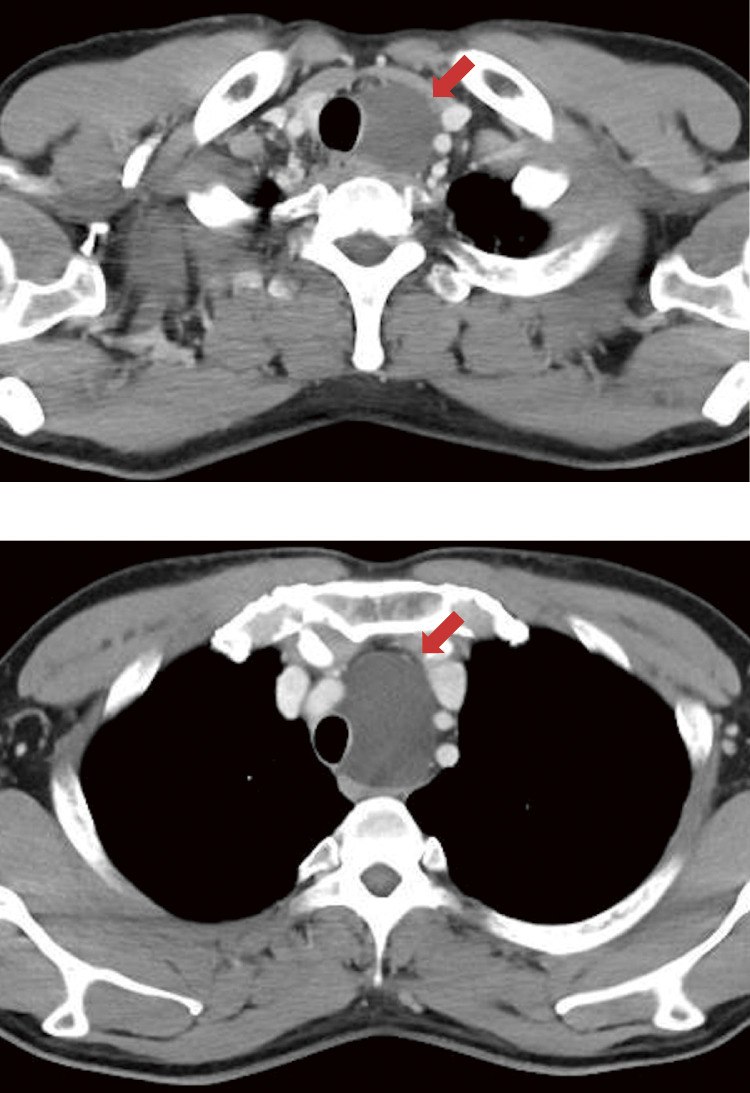
Computed tomography findings. Computed tomography reveals a cystic lesion (arrow) with a maximum diameter of 78 mm, extending from the superior to the middle mediastinum, compressing the trachea to the right.

**Fig. 3:**
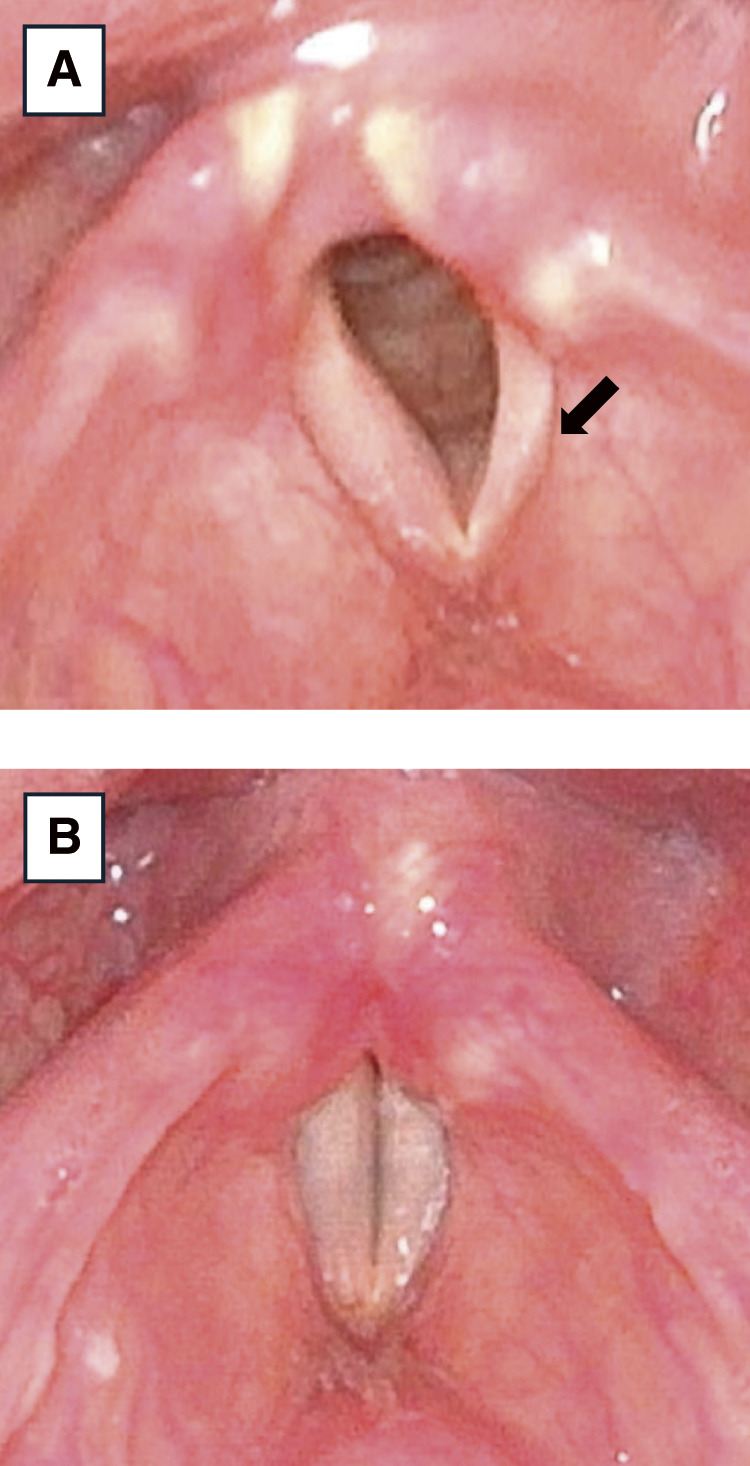
Laryngoscopy examination findings. (**A**) Laryngofiberscopy shows left vocal cord (arrow) paralysis and atrophy. (**B**) Laryngofiberscopy 2 months postoperatively shows improvement in left vocal cord paralysis and atrophy.

A 47-year-old man experienced hoarseness and dyspnea upon exertion for 1 month. A soft thumb-sized mass was palpable in the left anterior neck. Chest radiography revealed a mass on the left side of the trachea, causing tracheal deviation to the right (**Fig. 1A**). Computed tomography revealed a cystic lesion with a maximum diameter of 78 mm extending from the superior to the middle mediastinum and compressing the trachea to the right (**Fig. 2**). Pulmonary function tests indicated an obstructive ventilatory defect with an upper airway obstruction pattern on the flow-volume curve. Serum calcium levels were normal. Aspiration of the cyst revealed a clear serous fluid without any malignancy. Laryngofiberscopy revealed left vocal cord paralysis and atrophy, suggesting long-term left recurrent laryngeal nerve paralysis due to tumor compression (**Fig. 3A**).

**Fig. 4:**
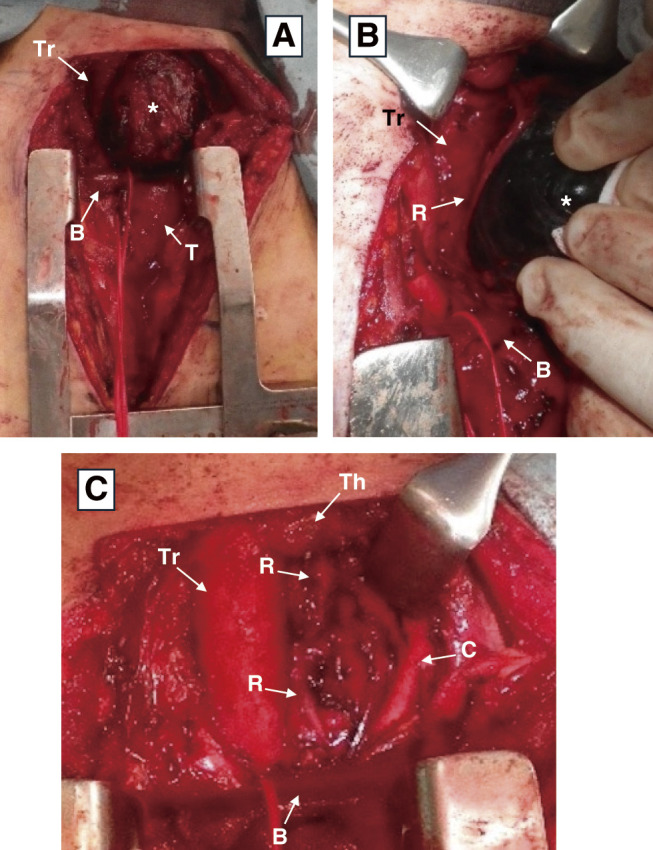
Surgical findings. (**A**) Thick-walled cystic tumor (asterisk) compresses the trachea to the right. (**B**) Left recurrent laryngeal nerve adhered to the right side of the tumor (asterisk). (**C**) The tumor was dissected from the surrounding tissue and excised. The left recurrent laryngeal nerve was dissected and preserved. *Tr, trachea; R, left recurrent laryngeal nerve; Th, left thyroid lobe; C, left common carotid artery; B, left brachiocephalic vein; T, thymus

Although a definitive preoperative diagnosis was not obtained, tumor resection was performed in collaboration with the departments of thoracic surgery and head and neck surgery for diagnostic and therapeutic purposes because mediastinal cystic tumors can cause tracheal deviation and recurrent laryngeal nerve paralysis (**Fig. 4**).

In the extended neck position, a transverse incision was placed 1 finger-width from the clavicular head. The left sternohyoid and sternothyroid muscles were dissected, revealing the lower pole of the left thyroid lobe and a caudally located cystic tumor with a thick wall. The tumor displaced the left thyroid lobe cranially and the trachea to the right, with mild adhesions to the surrounding tissues. The cranial aspect of the tumor was dissected along its wall and separated from the left thyroid lobe. Additionally, further dissection on the left side of the tumor separated it from the posterior surface of the left sternocleidomastoid muscle and left common carotid artery. Dissection was then performed between the posterior aspect of the tumor and the cervical vertebrae. Dissection on the right side of the tumor separated it from the trachea and posterior esophagus. The left recurrent laryngeal nerve, which adhered to the right side of the tumor and ran cranio-caudally, was identified and preserved. The tumor extended posteriorly to the left brachiocephalic vein, making it difficult to dissect solely from the neck. Thus, a skin incision was made at the anterior chest midline, and an upper three-quarters midline sternotomy was performed. Dissection was performed between the thymus and the pleura on both sides, and the left brachiocephalic vein was separated. Because of the strong adhesions between the cranial side of the tumor and the thymus, the cranial portion of the thymus was resected along with the tumor. Additionally, dissection from the caudal side of the tumor separated it from the left brachiocephalic vein and left common carotid artery on the left side and posterior esophagus and trachea on the right. The tumor was dissected from the recurrent nerve on the right side, preserving it, and excised without damaging the cystic wall. To avoid nerve damage, we performed sharp dissection and minimized tension on the nerve. We performed intraoperative nerve stimulation both during and after the dissection procedure. There was no response at either time point, and the presence of complete laryngeal nerve paralysis was identified; thus, we deemed intraoperative neuromonitoring redundant.

**Fig. 5:**
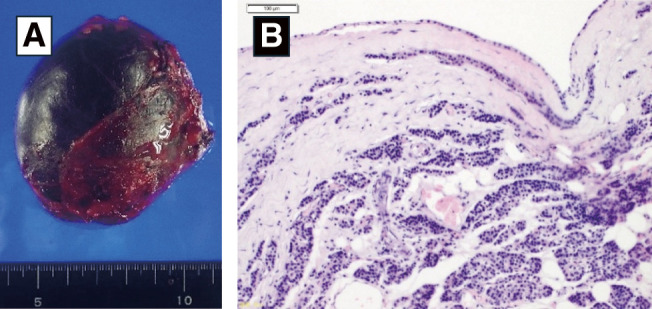
Pathological findings. (**A**) The resected specimen is seen as a 55 mm cystic tumor containing a brownish serous fluid. (**B**) Hematoxylin-eosin staining shows the cyst wall lined with cuboidal epithelium with a clear cytoplasm, leading to the diagnosis of a mediastinal parathyroid cyst.

The resected specimen was a 55 mm cystic tumor containing a brownish serous fluid. The cyst wall was lined with cuboidal epithelium with clear cytoplasm, which led to the diagnosis of a mediastinal parathyroid cyst (**Fig. 5**). Postoperatively, tracheal deviation improved rapidly (**Fig. 1B**). Two months postoperatively, the hoarseness had resolved. Laryngofiberscopy showed improvement in vocal cord movement and atrophy (**Fig. 3B**).

## DISCUSSION

Mediastinal parathyroid cyst is a rare cystic disease that involves the parathyroid tissue within its walls. These cysts are often found in the superior mediastinum and can be classified into functional types with hyperparathyroidism and non-functional types without hyperparathyroidism.^1)^ A mediastinal parathyroid cyst is usually asymptomatic; however, in rare cases, it can cause hoarseness due to recurrent laryngeal nerve paralysis, dysphagia, or tracheal compression-induced dyspnea.^1)^ Surgery is indicated when the cysts are functional, symptomatic, undiagnosed, suspected of malignancy, or recur after aspiration.^2)^

In this case, surgical resection of a mediastinal cystic tumor with recurrent laryngeal nerve paralysis and tracheal deviation led to the diagnosis of a mediastinal parathyroid cyst. Although parathyroid hormone levels were not measured preoperatively, normal serum calcium levels suggested that the cyst was likely non-functional. Of the 4 reported cases of mediastinal parathyroid cysts causing recurrent laryngeal nerve palsy,^3–6)^ only one stated an improvement in recurrent laryngeal nerve palsy following surgery,^3)^ and only one other was accompanied by tracheal deviation.^6)^ To the best of our knowledge, the current case is the first report of improvement in both recurrent laryngeal nerve paralysis and tracheal deviation following the resection of a mediastinal parathyroid cyst.

Laryngofiberscopy revealed atrophy of the left vocal cord, suggesting long-term left recurrent laryngeal nerve paralysis, which improved postoperatively. Due to tumor-induced compression, the recurrent laryngeal nerve developed localized circulatory impairment, resulting in paralysis. However, since this was limited to a transient, reversible conduction disturbance, it was considered to have improved following tumor removal, although 2 months were required for full recovery after surgery. Long-term recurrent laryngeal nerve paralysis can improve with decompression, emphasizing the need for proactive surgical intervention.

Additionally, even if there is no response to intraoperative nerve stimulation, as observed in this case, improvement of paralysis is still possible. This necessitates careful preservation of the recurrent laryngeal nerve.

Further case studies are required to better understand the efficacy of surgical interventions in mediastinal parathyroid cysts leading to recurrent laryngeal nerve paralysis and tracheal deviation.

## CONCLUSIONS

This reported case of a mediastinal parathyroid cyst showed improvement in both recurrent laryngeal nerve paralysis and tracheal deviation after surgical resection. Even in cases where long-term recurrent laryngeal nerve paralysis is suspected and there is no response to intraoperative nerve stimulation, improvement is still possible. Therefore, proactive surgical resection and careful preservation of the recurrent laryngeal nerve are necessary.

## ACKNOWLEDGMENTS

We would like to thank Editage (www.editage.jp) for editing the English language.

## DECLARATIONS

### Funding

This study received no funding.

### Authors’ contributions

MiS wrote the article.

MiS, HK, MaS, HY, and TT performed surgical procedures.

All the authors discussed the contents of the manuscript.

TT supervised manuscript editing.

All the authors have read and approved the final version of the manuscript.

### Availability of data and materials

Data sharing is not applicable to this article as no datasets were generated or analyzed during the study.

### Ethics approval and consent to participate

Patient privacy was considered, and the manuscript did not include any identifying information. Informed consent was obtained from the patient for the use of the patient’s clinical data and accompanying images.

### Consent for publication

Informed consent was obtained from the patient for publication of the patient’s clinical data and accompanying images.

### Competing interests

The authors have no competing interests to declare.
